# Effect of Distress, Anxiety, and Depressive Symptoms on SARS-CoV-2 mRNA BNT162b2 Vaccine Efficacy in Cancer Patients

**DOI:** 10.3390/cancers16234012

**Published:** 2024-11-29

**Authors:** Gabriella Rondanina, Tania Buttiron Webber, Oriana D’Ecclesiis, Marco Musso, Irene Maria Briata, Nicoletta Provinciali, Monica Boitano, Matteo Clavarezza, Mauro D’Amico, Carlotta Defferrari, Alberto Gozza, Leonello Innocenti, Alessio Carbone, Martino Oliva, Emanuela Marcenaro, Francesca Filauro, Sara Gandini, Andrea DeCensi

**Affiliations:** 1 Oncology Department, E.O. Ospedali Galliera, 16128 Genoa, Italy; gabriella.rondanina@gmail.com (G.R.); irene.maria.briata@galliera.it (I.M.B.); nicoletta.provinciali@galliera.it (N.P.); monica.boitano@galliera.it (M.B.); matteo.clavarezza@galliera.it (M.C.); mauro.damico@galliera.it (M.D.); carlotta.defferrari@galliera.it (C.D.); alberto.gozza@galliera.it (A.G.); leonello.innocenti@galliera.it (L.I.); alessio.carbone@galliera.it (A.C.); martino.oliva@galliera.it (M.O.); francesca.filauro@galliera.it (F.F.); andrea.decensi@galliera.it (A.D.); 2 European Institute of Oncology IRCCS, 20141 Milan, Italy; orianadec94@gmail.com (O.D.); sara.gandini@ieo.it (S.G.); 3 IST Nord-Centro Risorse Biologiche, Policlinico San Martino, 16132 Genoa, Italy; marco.musso@hsanmartino.it; 4 Department of Experimental Medicine (DIMES), University of Genoa, 16126 Genova, Italy; 5 IRCCS Ospedale Policlinico San Martino, 16132 Genova, Italy; 6 Wolfson Institute of Preventive Medicine, Queen Mary University of London, London E1 4NS, UK

**Keywords:** COVID-19 vaccines, immunogenicity, vaccine, neoplasms, psychological distress, depressive disorder, anxiety disorders, C reactive protein, D dimer

## Abstract

Background/Objectives: Our previous study of 291 cancer patients, we showed that 20% did not respond to two doses of COVID-19 vaccine administered six weeks apart. Methods: Here, we investigated if psychological factors (distress, anxiety, and depressive symptoms) affected antibody response and markers of vaccine activation (D-dimer) after 6 months from initial vaccination. Results: Overall, 31 subjects (14.2%) had no antibody response at 6 months. Our analysis revealed significant predictors of vaccine failure, including the stage of metastatic disease and high-stress levels (OR = 2.46, 95% CI, 1.05–5.77, *p* = 0.04). Notably, nonresponders showed twice the prevalence of distress than responders (21% vs. 10%, *p* = 0.04). Longitudinal measurements of IgG levels indicated that participants with high depressive symptoms at baseline maintained lower antibody levels over six months (*p* = 0.003). In addition, women with high anxiety showed reduced levels of D-dimer at 6 months (*p* = 0.03). These data also showed that smokers and former smokers had significantly lower antibody levels than their nonsmoking counterparts (*p* = 0.0004). At baseline, the high discomfort rate (≥5) was 34.4% in women and 23.8% in men; only men experienced an increase in median discomfort during the observation period. Moreover, a higher educational level was related to increased distress among women (*p* = 0.046). Conclusions: These findings underscore a critical association between elevated psychological distress and reduced immune responses to the COVID-19 vaccine, emphasizing the urgent need for targeted psychological and behavioral support within this vulnerable population.

## Simple Summary

This study aimed to address an urgent issue: about 20% of cancer patients undergoing treatment do not respond adequately to the standard two-dose COVID-19 vaccination. This highlights the need to identify factors influencing vaccine efficacy in this vulnerable group, including psychological conditions such as distress, anxiety, and depression. The primary objectives were to determine whether these psychological factors, along with clinical variables such as metastatic stage and smoking, influenced antibody response and vaccine activation six months post-vaccination. This study also examined changes in psychological distress over time in relation to gender and education. The results showed that 14.2% of patients did not achieve an antibody response at six months, with high distress and metastatic cancer as significant predictors of non-response (OR = 2.46, *p* = 0.04). These findings suggest that integrating distress management strategies into oncology care could enhance vaccine response, supporting improved health outcomes for cancer patients.

## 1. Introduction

The SARS-CoV-2 pandemic has disproportionately impacted cancer patients, with evidence linking the virus to significantly increased morbidity and mortality in this vulnerable population [1,2]. This heightened risk underscores the critical need for effective preventive interventions, such as vaccination, to mitigate the severe outcomes of COVID-19. While clinical and biological determinants of vaccine efficacy are well-recognized, the role of psychological and behavioral factors remains less explored. Emerging evidence highlights that psychosocial factors can profoundly influence immune responses, including the production of antibodies following vaccination against COVID-19 [3,4,5,6]. Specifically, elements such as social cohesion have been implicated in modulating antibody responses to the COVID-19 vaccine [7]; however, the extent to which psychological factors affect vaccine immunogenicity in immune-compromised cancer patients undergoing active treatment is still unknown.

In this study, we investigate the relationship between psychological distress, anxiety, and depressive symptoms and the immunogenicity of the BNT162b2 SARS-CoV-2 vaccine in cancer patients undergoing diverse treatments. We assessed immunogenicity by measuring rates of anti-spike immunoglobulin G (IgG) antibody positivity, aiming to identify psychological predictors of poor seroconversion—a widely accepted proxy for reduced vaccine efficacy [8]. Additionally, we evaluated D-dimer levels as a marker of vaccine activation. D-dimer, a fibrin degradation product, serves as an important prognostic biomarker associated with severe COVID-19 outcomes [9] and vaccine-induced thrombocytopenia and thrombosis [10]. Its elevation reflects the activation of the coagulation cascade and offers critical insight into the interplay between vaccination and coagulation pathways in cancer patients.

Finally, recognizing documented sex differences in psychological responses—where men often exhibit escalating distress during follow-up compared with women, whose levels tend to stabilize [11]—we explored whether sex-specific variations influence psychological distress and vaccine immunogenicity in this cohort. Through this comprehensive approach, our study seeks to elucidate the complex interactions between psychological health, biological responses, and vaccine efficacy in an at-risk population, offering new perspectives for optimizing vaccination strategies in oncology settings.

## 2. Materials and Methods

### 2.1. Study Design and Participants

Between March and July 2021, we conducted a prospective, observational cohort study to identify predictors of poor antibody response to the SARS-CoV-2 mRNA vaccine BNT162b2 (Pfizer-BioNTech) in cancer patients, with a specific focus on psychological factors. In a previous analysis conducted over the same period addressing clinical predictors, we demonstrated that chemotherapy, targeted therapy, hormone therapy, lymphopenia (<1 × 10^9^/L), and advanced age were associated with poor seroconversion (vaccine failure) in approximately 20% of patients after two doses of BNT162b2 [8].

The primary study design and patient characteristics have been described in detail previously [8]. Briefly, this study aimed to evaluate the antibody titer reactogenicity to the BNT162b2 vaccine in cancer patients undergoing active treatment. Inclusion criteria were: patients aged ≥18 years with an active malignancy, ongoing treatment or treatment completed within the past six months, and lymphocyte counts ≥ 0.5 × 10^9^/L (500/μL) (Supplemental Table S1). This threshold was established to exclude patients at heightened risk of infections due to chronic immunosuppressive therapy with lymphopenia < 0.6 × 10^9^/L [12].

Patients were categorized based on their most recent systemic therapy (chemotherapy, hormone therapy, biological therapy, or immunotherapy), and those who had not received treatment within 180 days before vaccine administration were classified as untreated.

Participants underwent clinical evaluations and blood sample collections at four predefined time points: (1) baseline prior to the administration of the first vaccine dose [Visit 1], (2) 21 days following the first dose [Visit 2], (3) 42 days post-baseline [Visit 3], and (4) six months after baseline [Visit 4]. This study was registered on ClinicalTrials.gov (ID: NCT04932863) and received ethical approval from the National Institute for Infectious Diseases in Rome as well as the local Ethical Committee. Recruitment occurred at Galliera Hospital in Genoa between 15 March and 21 July 2021, with all participants providing written informed consent.

### 2.2. Procedures

The vaccine treatment consisted of 30 μg of BNT162b2 (0.3 mL volume per dose) delivered in the deltoid muscle in 2 doses, 21 days apart. We pooled treatments in five groups to facilitate comparisons: active surveillance (no treatment), chemotherapy, hormone therapy, targeted therapy/monoclonal antibodies, and immune checkpoint inhibitors.

At the beginning of the clinical visit, the distress thermometer in the previous week was compiled as previously described [13]. The National Comprehensive Cancer Network (NCCN) recommends that all cancer survivors undergo distress screening as a “sixth vital sign” to mitigate the risk of developing severe psychological conditions such as anxiety, depression, and impaired coping mechanisms [14]. The distress screening tool is a self-reported, single-item measure utilizing a 0 (no distress) to 10 (extreme distress) Likert scale, designed to resemble a thermometer.

The patients were also asked to identify the sources of their distress using a 39-item checklist that encompasses various domains, including emotional, physical, practical, familial, and spiritual/religious challenges. According to the NCCN guidelines (2013), distress scores are categorized into two levels: low (0–4) and high (5–10) [15].

The Distress Thermometer (DT) has demonstrated robust reliability and has been widely translated and validated across multiple languages, including Italian [16].

Anxiety and depressive symptoms were evaluated using the Hospital Anxiety and Depression Scale [17]. The scoring system for both anxiety and depressive symptoms was 0–7 = Normal, 8–10 = Borderline elevated, and 11–21 = Elevated. The antibody titer was quantified using the LIAISON^®^ SARS-CoV-2 S1/S2 IgG assay, a chemiluminescent immunoassay (CLIA) designed to measure IgG antibodies targeting the S1/S2 dimeric domains of the SARS-CoV-2 spike protein in human serum [18,19]. The system reports SARS-CoV-2 S1/S2 IgG concentrations in arbitrary units per milliliter (AU/mL) with a detection range of 3.8–400 AU/mL, providing graded results based on these measurements. The threshold of seroconversion was ≥25 AU/mL according to our lab procedures because of a previous preliminary study of the correlation between the level of antibodies and concomitant T-cell response that further proved immunization.

During this study, a more sensitive LIAISON^®^ SARS-CoV-2 Trimeric Spike IgG assay expressed in BAU/mL [20] became available and was introduced at 6 months and compared with the dimeric method.

Since there was no significant difference in the proportion of non-responders at 6 months between the dimeric and trimeric detection methods (<25 AU/mL and 33.8 BAU/mL, respectively (Supplemental Table S2)), results of the primary endpoint at 6 months were calculated with the cut-off of the more sensitive trimeric method [20].

D-dimer was measured with an automated, latex-enhanced turbidimetric immunoassay [HemosIL^®^ D-Dimer HS 500, Instrumentation Laboratory (IL), as previously described [21]. The primary objective was to assess if psychological factors, including distress, anxiety, and depressive symptoms, were associated with poor antibody titer reactogenicity (cut-off levels <25 AU/mL or <33.8 BAU/mL) to BNT162b2 vaccine at 6 months (primary endpoint). The secondary endpoints were the repeated measure analysis of antibodies measured with the dimeric method at 6 months and the effect of psychological variables on biomarkers of vaccine response such as D-dimer.

### 2.3. Statistical Analysis

The continuous variables were summarized using the median and interquartile range (IQR), while categorical variables were described using absolute and relative frequencies.

Depending on the type of data, Fisher’s exact test, Wilcoxon rank-sum test, or Kruskal–Wallis rank-sum test were applied for statistical analysis. Multivariable logistic regression was applied to identify independent factors associated with vaccine failure at 6 months. Multivariable mixed effects models for repeated measures analysis were adopted both to analyze changes in time of IgG response, D-dimer, and DT and to identify independent factors associated with these outcomes. The normal distribution of residuals from fully adjusted models was graphically checked and log transformation was adopted when it was needed to achieve normality. The odds ratio (OR) and percentages of IgG non-responders are presented with 95% confidence intervals.

Distress was categorized as ‘High’ or “Low”, considering 5 as the cut-off point. Bar plots were presented to describe the percentages of responders by type of distress. Independent variables were included in the model, collapsing the categories when the frequencies were too low (e.g., stage IV vs. I–III) and categorizing continuous variables such as age, considering the median value, to have more clinically interpretable estimates.

Boxplots were generated to compare patient characteristics in terms of change in outcomes in time from baseline to 6 weeks and 6 months.

All *p*-values were two-sided with a 5% significance level. The analyses were carried out using the R studio (R version 4.2.3) software.

## 3. Results

From 15 March 2021 to 21 July 2021, 407 patients were screened for vaccination and offered to participate in this study, of whom 320 agreed to participate. Of these, 291 were assessable at 42 days, and 218 remained assessable at 6 months due to loss to follow-up or death, as shown in the CONSORT statement in Supplemental Figure S1.

**Figure 1:**
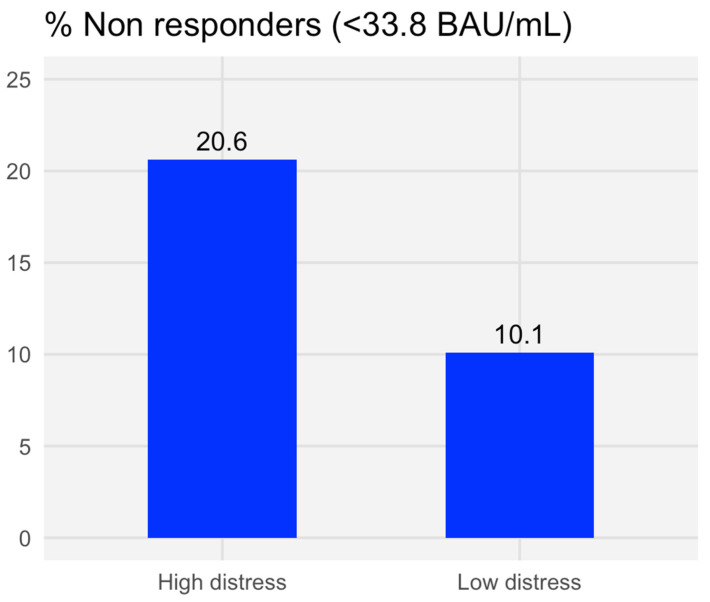
Number (%) of non-responders according to antibody measurement at 6 months by distress level at baseline.

The main subject and tumor characteristics of the 218 patients are summarized in Table 1. The median age was 68.2 years, approximately 60% were females and had metastatic disease, over 20% were treated >6 months ago, one-third were on (neo)adjuvant treatment, and two-thirds were on 1st–3rd line of treatment. Overall, 31 subjects (14.2%) had no antibody response at 6 months. The predictors of vaccine failure at 6 months are summarized in Table 2. In addition to the metastatic disease stage, the only independent significant variable was high distress (OR = 2.46, 95% CI, 1.05–5.77, *p* = 0.04, Table 2). The proportion of non-responders was similar between the dimeric and the most sensitive trimeric assay method (Supplemental Table S2). Among non-responders, the proportion of subjects with high distress versus low distress was twofold higher (21% vs. 10%, respectively, *p* = 0.04, Figure 1).

**Figure 2:**
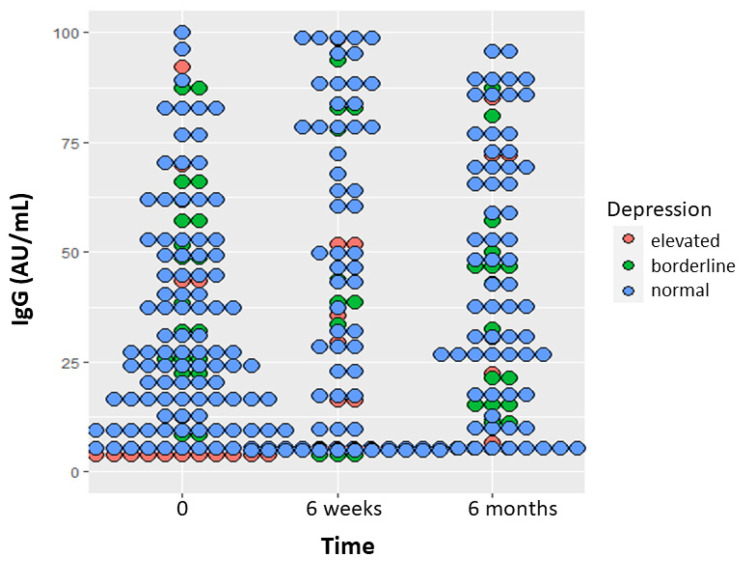
Dotplot showing repeated measure analysis of IgG (AU/mL) response according to depressive symptoms level at baseline.

Repeated measure analysis of the IgG response dotplot measured with the dimeric method at 0, 6 weeks, and 6 months indicates that participants with high depressive symptoms level at baseline had lower antibody response during the 6-month time (*p* = 0.003, Figure 2). In addition, women with elevated anxiety levels at baseline had lower D-dimer levels at 6 months (*p* = 0.03. The proportion of high distress remained stable during the 6-month observation period: 25.9% at baseline, 24.8% at 6 weeks, and 25.0% at 6 months. At baseline, women with high distress were 34.4% versus 23.8 in men (*p* = 0.08). The median level of distress in women was two at baseline and did not change in the 6-month observation period, whereas it increased from one to two in men (Supplemental Figure S2). Repeated measure analysis showed that high distress at baseline (*p* = 0.0001) and higher education (*p* = 0.04) explained high distress at 6 months (Supplemental Figure S3), but this effect was limited to women only (*p* = 0.046).

As a sensitivity analysis, we also assessed COVID antibodies as a continuous variable, but we did not find any significant association with distress (*p* = 0.93 and 0.27 with AU and BAU, respectively). We also tried to assess the association of smoking with response to the vaccine, including the variable in the model, but it was not significant, and the results did not change. 

No significant associations between high and low psychological symptoms and cancer sites were found (Supplemental Table S3).

## 4. Discussion

Cancer patients are at an increased risk of severe COVID-19 with higher mortality rates [1,2], positioning them as a highly vulnerable population warranting prioritized access to vaccination. Furthermore, their immunogenic response to SARS-CoV-2 infection is lower compared with the general population [22], suggesting a potentially diminished vaccine response. This contrasts with the robust 95% efficacy observed in healthy individuals following administration of the BNT162b2 vaccine [23]. Our initial report showed that chemotherapy, targeted therapy/monoclonal antibodies, hormone therapy, lymphocyte count <1 × 10^9^/L, and increasing age predicted poor seroconversion at 6 weeks after two doses of BNT162b2 in up to 20% of patients [8], indicating the need for additional vaccine doses and long-term follow-up.

The present study was designed to determine if psychosocial factors predicted failure of the BNT162b2 vaccine at 6 months in a cohort of patients under treatment for solid cancers to guide better strategies to increase vaccine efficacy in non-responders. Our main finding indicates that a high level of psychological distress is associated with a 20% failure rate of the COVID-19 vaccine versus 10% in patients with low distress, a statistically significant difference.

Interestingly, patients with depressive symptoms demonstrated a lower D-dimer response to vaccination. Elevated plasma D-dimer levels, a marker of coagulation cascade activation following vaccination, are associated with poorer outcomes in COVID-19 patients [9,24]. This suggests that depressive symptoms may impair the physiological response to vaccination.

The concept of psychological stress has been studied for a long time in the field of psycho-oncology for its repercussions not only on the quality of life of cancer patients but also on their response to treatment and side effects; however, it is important to consider its connection with the affective experiences and the emotional discomfort that are expressed in these stressful events. Several studies highlight the influence of emotional aspects on the functioning of the immune system and, consequently, the antibody response to vaccines, including the COVID-19 vaccine. A lack of social cohesion, combined with feelings of loneliness, has been observed to affect immune function negatively [7,25,26]. A meta-analysis revealed a significant negative association between psychological stress and antibody responses to influenza vaccination [4]. Moreover, prior research suggests that psychological and behavioral interventions can improve vaccine responsiveness, including COVID-19 vaccine [3]. Individuals with depressive symptoms have been shown to have a lower antibody response to COVID-19 vaccination [27], and patients with mental disorders, including major depressive symptoms, have lower responses to vaccination in general [28].

The nocebo effect, a phenomenon wherein negative expectations amplify perceived side effects, may also play a critical role in modulating vaccine responses. As described by Amanzio et al. [29], heightened public anxiety and negative discourse surrounding vaccination can lead to the misattribution of common symptoms, such as fatigue or mild fever, to adverse vaccine effects. This amplification of perceived side effects could further exacerbate psychological distress, potentially reducing vaccine acceptance and adherence. In this context, addressing the nocebo effect through evidence-based communication emphasizing findings from placebo-controlled trials can mitigate unwarranted concerns, bolster trust, and support vaccination efforts.

In our study, the association between high distress and vaccine failure, high anxiety and decreased D-dimer, elevated depressive symptoms, and lower antibody response can represent and offer an objective and biological measurement of what is happening at an organic level in situations of psychological fragility and vulnerability. Since the antibody response or the increase in biomarkers of vaccine activation is lower in people who have high distress, high anxiety, and high depressive symptoms, our findings strengthen the relationship between the mind and the immune system. In some studies, it is observed that a positive mental attitude in dealing with the disease has the function of a ‘modulator of the immune system’ and is associated with a greater immune response [30]. In the same way, a healthy lifestyle is beneficial for health and, therefore, favors a better functioning of the immune system [31,32]. These mechanisms describe a general psychic and organic attitude towards life. Psychological stress and depressive symptoms can significantly impair immune system function, leading to reduced antibody responses and overall diminished physiological activation. This underscores the necessity of addressing the emotional well-being of cancer patients to enhance their immune responses, including vaccine efficacy. Recent data indicate that psychosocial interventions, such as cognitive-behavioral therapy (CBT), can improve immune function by decreasing pro-inflammatory cytokines and increasing immune cell counts [33]. Additionally, studies have shown that stress and depression are associated with immunosuppression in cancer patients, highlighting the importance of managing these psychological factors to support immune health [34]; therefore, integrating psychological care into the treatment of cancer patients is crucial for fostering life-oriented attitudes and enhancing vaccine efficacy.

Our findings suggest no significant change in psychological distress over the 6-month vaccine exposure, at variance with prior studies suggesting an amelioration of distress after the COVID-19 vaccine in the general population [35], possibly because in our population’s advanced-stage disease is a much higher distress source than COVID-19. In contrast with a previous study [12], we did not find a significant interaction between sex and time of distress level 6 months apart, but only a small trend to a worsening in males. This may be related to a shorter time exposure compared with our prior observation 16 months apart [12]. Interestingly, women with a high educational level were those at higher risk of elevated distress after 6 months, consistent with the finding of a large cohort where highly educated persons are at higher risk of distress than those with medium/low educational levels after adjustment for confounders [36].

An important limitation of this study is the moderate sample size, which precluded stratification by cancer type and treatment modality. Nonetheless, the findings underscore the importance of integrating psychological care with oncological treatment to foster a life-oriented attitude and bolster vaccine efficacy. This is especially crucial given the observation that advanced-stage disease, rather than COVID-19 itself, remains the primary source of distress in our cohort, contrasting with trends observed in the general population [32]. Addressing these multifaceted challenges through holistic, evidence-based interventions can significantly improve health outcomes in this vulnerable population.

## 5. Conclusions

This study demonstrates that psychosocial factors, such as psychological distress, depressive symptoms, and smoking status, significantly impact the immune response to the BNT162b2 COVID-19 vaccine in cancer patients. High distress levels were associated with a 20% vaccine failure rate, while depressive symptoms correlated with reduced physiological vaccine activation. Smoking was linked to lower antibody titers, emphasizing the immunosuppressive effects of tobacco use.

The findings highlight the importance of addressing psychological well-being and lifestyle factors to enhance vaccine efficacy in this vulnerable population. Evidence-based communication strategies are essential to counteract the nocebo effect, reduce anxiety, and improve vaccine acceptance. A holistic approach integrating psychosocial support, lifestyle interventions, and targeted medical care is critical for optimizing outcomes in cancer patients. Future research should explore psychosocial interventions and stratify analyses by cancer type and treatment to deepen understanding of vaccine response.

## Figures and Tables

**Table 1:** Main subject characteristics at 6 months (N = 218).

Age, Years	67.6 [58.5–74.3]
Sex	
Female	135 (61.9)
Male	83 (38.1)
Body Mass Index, kg/m^2^	24.6 [22.4–27.9]
Tumor site	
Digestive	64 (29.4)
Lung	25 (11.5)
Breast	57 (26.1)
Genitourinary and gynecologic	66 (30.3)
Other *	6 (2.7)
Stage	
I	17 (7.8)
II	46 (21.1)
III	33 (15.1)
IV	122 (56.0)
Line of treatment	
Adjuvant/Neoadjuvant	89 (40.8)
1st	75 (34.4)
2nd	32 (14.7)
3rd or more	21 (9.6)
Missing	1 (0.5)
Type of treatment	
No treatment **	54 (24.8)
Chemotherapy	71 (32.6)
Hormone therapy	57 (26.1)
Biological therapy	21 (9.6)
Immunotherapy	15 (6.9)

* Other includes 5 head and neck cancer, 2 choroid melanoma, 2 Chronic lymphocytic leukemia CLL, 1 multiple myeloma, 1 brain glioma. ** Patients with last treatment ≥180 days before the vaccine administration were considered as untreated; ICI, immune checkpoint inhibitors.

**Table 2:** Predictors of no antibody response at 6 months.

	Method	
	Dimeric AU ^1^ OR (CI 95%), *p*	Trimeric BAU ^2^ OR (CI 95%), *p*
Age, years		
≤68.2 vs. >68.2	0.58 (0.17–1.82), 0.36	0.51 (0.21–1.19), 0.12
Stage		
Stage IV vs. I–III	4.52 (1.32–20.9), 0.03	2.80 (1.15–7.56), 0.03
Distress at baseline *		
High vs. low	3.77 (1.24–12.2), 0.02	2.46 (1.05–5.77), 0.04

* High distress threshold ≥ 5. ^1^ Analytical Ultracentrifugation (AU); ^2^ Biophysical Analytical Ultracentrifugation (BAU).

## Data Availability

Individual participant data are not publicly available because this requirement was not anticipated in this study protocol. Tania Buttiron Webber, Andrea DeCensi, Oriana D’ecclesiis, and Sara Gandini had full access to all the data in this study and took responsibility for the integrity of the data and the accuracy of the data analysis. Data may be shared upon request for collaborative studies.

## Supplementary Materials

The following supporting information can be downloaded at https://www.mdpi.com/article/10.3390/cancers16234012/s1, Figure S1. Participant flow diagram; Figure S2. Pre and post-vaccination level of distress according to sex; Figure S3. Dotplot of repeated measure analysis of distress according to educational level; Table S1. Inclusion criteria: Table S2. Comparison of vaccine non-responders between assay methods; Table S3. Univariable analyses describing the association between high and low psychological symptoms and cancer sites.
