# Supplementary material for: Effect of Distress, Anxiety, and Depressive Symptoms on SARS-CoV-2 mRNA BNT162b2 Vaccine Efficacy in Cancer Patients
Source: Cancers (Basel). 2024 Nov 29;16(23):4012. doi: 10.3390/cancers16234012 (PMC11640422; doi:10.3390/cancers16234012)
Supplement: Supplementary file 1 [file cancers-16-04012-s001.zip › cancers-3308929-supplementary.pdf]

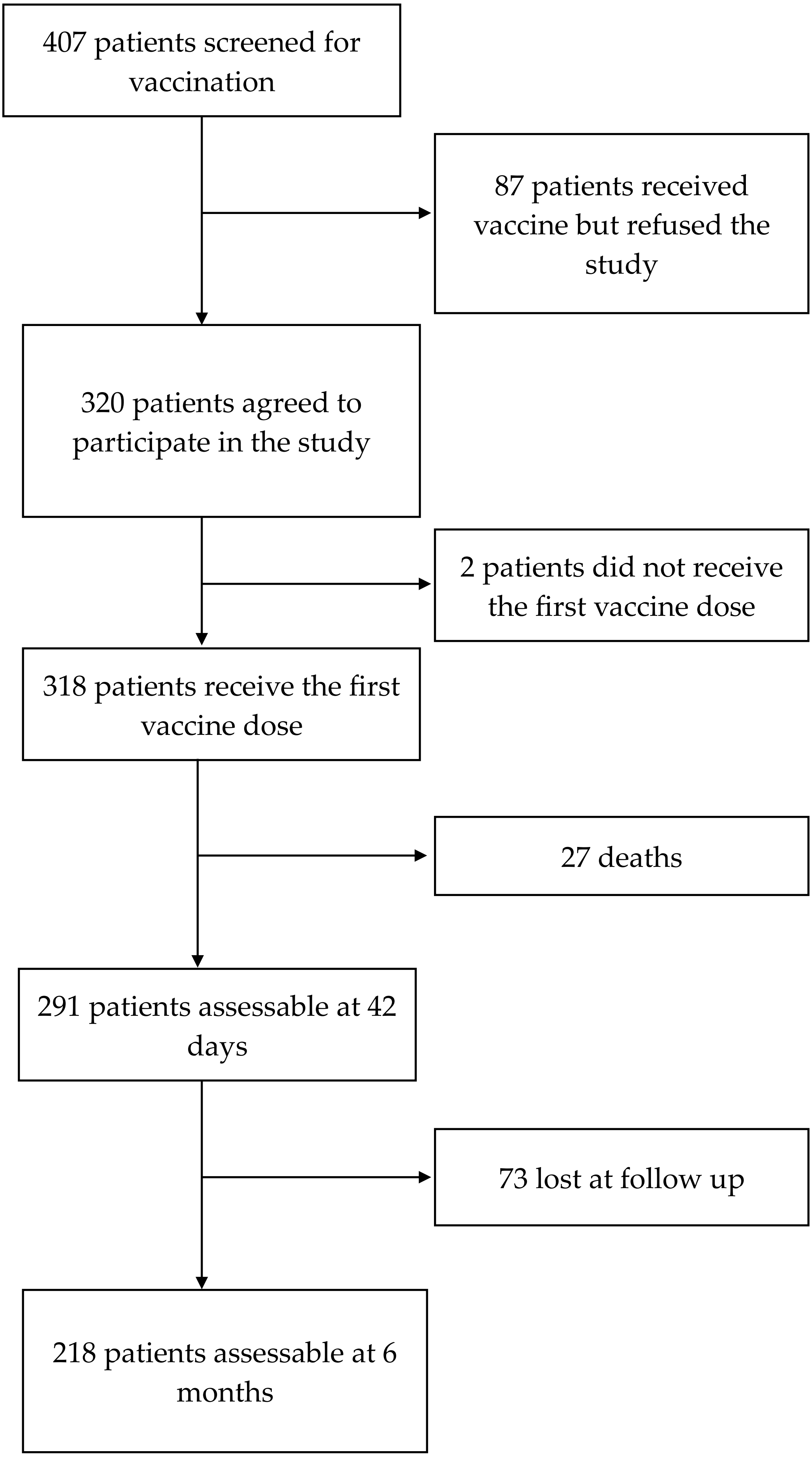

Supplemental Figure S1. Participant flow diagram

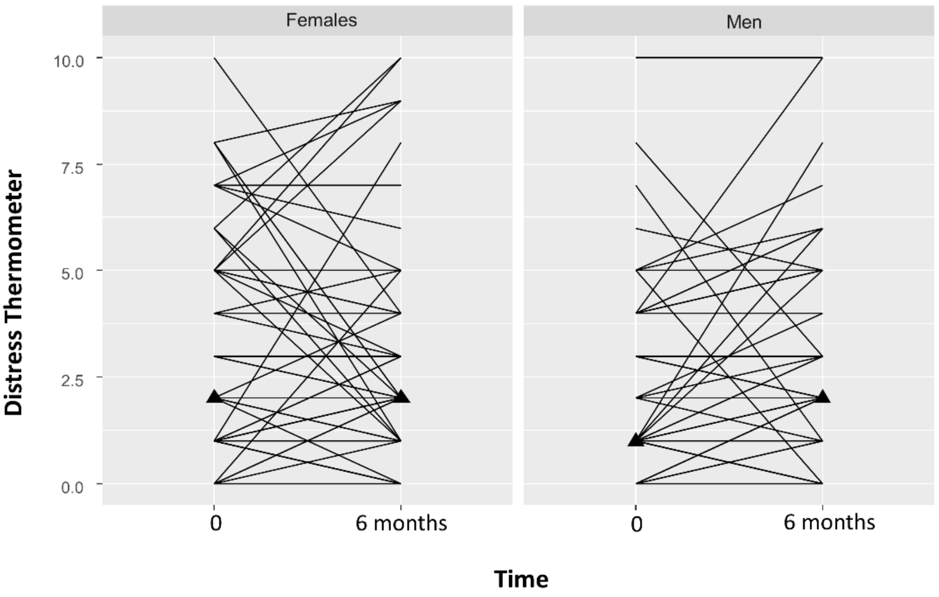

Supplemental Figure S2. Pre and post vaccination level of distress according to sex

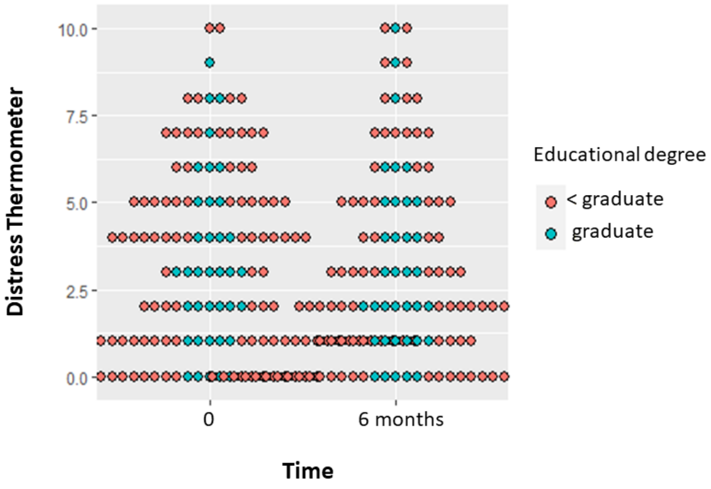

Supplemental Figure S3. Dotplot of repeated measure analysis of distress according to educational level

Supplemental Table S1. Inclusion criteria

**Inclusion Criteria**
Patients with malignancy
Aged ≥18 years
Ongoing treatment or treatment completed within the past six months
Lymphocytes count ≥0.5 × 109/L (500/μL) based on the risk of infections in subjects on chronic immunosuppressive therapy with lymphopenia <0.6 × 109/L

Supplemental Table S2. Comparison of vaccine non-responders between assay methods

	**Dimeric AU**[1] (N = 175)	**Trimeric BAU**[2] (N = 218)	*p value*
**Non responders**	18 (10.2)	31 (14.2)	0.31
**Responders**	157 (89.8)	187 (85.8)	

n (%)

[1]Analytical Ultracentrifugation (AU)
[2]Biophysical Analytical Ultracentrifugation (BAU)

Supplemental Table S3. Univariable analyses describing the association between high and low psychological symptoms and cancer sites.

	**High** n (%)	**Low** n (%)	*p value*
	80 (30.1)	186 (69.9)	
**Anxiety**, by Tumor site			
Digestive	7 (41.2)	10 (58.8)	0.68
Lung	17 (32.1)	36 (67.9)	
Breast	10 (25.6)	29 (74.4)	
Genitourinary and gynecologic	46 (29.3)	111 (70.7)	
Other	46 (29.3)	46 (29.3)	
**Depressive symptoms**, by Tumor site			
Digestive	19 (32.2)	40 (67.8)	0.27
Lung	37 (35.9)	66 (64.1)	
Breast	17 (26.1)	48 (73.9)	
Genitourinary and gynecologic	4 (21.0)	15 (79.0)	
Other	3 (15.0)	17 (85.0)	
**Distress**, by Tumor site			
Digestive	29 (31.9)	62 (68.1)	0.50
Lung	9 (32.1)	19 (67.9)	
Breast	23 (35.9)	41 (64.1)	
Genitourinary and gynecologic	17 (23.6)	55 (76.4)	
Other	2 (18.2)	9 (81.8)	
